# Case of Hæmatemesis with Melæna

**Published:** 1809-09

**Authors:** E. Campbell


*Case of Hæmatemesis with Melæna.* Communicated by Dr. E. Campbell.

——— Le Clerc, a Frenchman, æt. 40, asthmatic, and of a spare habit, butler at the time to the Right Hon. H. Grattan, was severely attacked by a vomiting of blood attended tended with syncope, in the night and morning of the 29th and 30th of May last. The first attack appears to have been preceded by, and accompanied with, a fit of coughing, but without any previous observable sense of oppression or fulness about the præcordia. Three weeks before this, he fell by accident down some steps into the area: not feeling however much inconvenience from the fall, he neglected it, and continued his occupations until the evening of the attack; having had on the same day a natural stool.

Early next morning, the 30th, Mr. Tegart of Pall-Mall, and myself, saw him for the first time. He had been using some medicines in the night. The quantity of blood now thrown up, into different vessels, and on the bed and floor, was probably not less than from 10 to 12 pounds. When recovered in some degree from the death-like state of lipothymia, in which we found the patient, he complained of extreme debility, vertigo, great flatus, head-ache, and thirst; his pulse was about 80, soft but unsteady.

Removed into a more airy chamber, he now began to take the cerussa acetata with opium in pills. His bowels were kept freely open by laxative emulsions, cooling injections, vitriolated magnesia, and crystal. tart. severally employed according to circumstances. The stools thus procured were at first scanty, and pitch-like, being black, and somewhat indurated; towards evening they became liquid and copious, with a large proportion of *bloody* fluid: from this time, they continued to the last, more or less fluid; always quite black and fœtid; sometimes glossy, exactly like pitch; seldom figured, and apparently but little real alvine fæces in them.

After an interval of 28 hours, he had another attack of Hæmatemesis, (slight in comparison) when he threw up about a pound of red blood, the paroxysm ending in fainting. After this, he never had any more return of vomiting of blood.

Next morning, that is, the third from the first attack, ten grains in all of the cerussa acetata had been taken; the dose having been increased from half a grain every 4th, to a grain and a half every 6th hour: the pills were then entirely discontinued.

He complained of no abdominal pains; slept at intervals; and continued taking in light nourishment and cooling drink, with a little wine and water; urgente lipothymia, which latter generally, more or less, accompanied his stools. Occasional nausea was relieved by saline mixtures. His tongue remained moist; but he had considerable thirst, and seldom any appetite for food. His pulse becoming daily more frequent, smaller, and intermitting, with other marks of increasing debility, he began to complain of amaurosis. His stools continued without any favourable change, very fœtid and atramental.

On the 7th of June, he complained of increasing dyspnœa; he had been restless and delirious in the night; his fluttering pulse was now upwards of 130; his urine continued natural from the first.

Next day, he lost the power of deglutition; and the following morning, the 9th, yielded to his fate.

The circumstances of this case rendering an inspection after death very desirable, the body was carefully opened by Mr. H. Earle of Hanover-Square. The spleen, not larger than natural, was found remarkably pale, soft, and easily broken, adhering to the diaphragm and cardia; and free of any obstruction.

The stomach on being opened, gave out a considerable quantity of fœtid gas; it was otherwise perfectly *empty*. On the inner face of the great curvature, was seen a circular *erosion* in and through the villous coat, barely a fourth of an inch in diameter, exposing to view an opening in the side of a large branch of the gastro-epiploic artery, communicating directly with the cavity of the stomach. A large probe was readily passed from the opening in the eroded spot, into the circular lumen of the artery, for some length in both directions: the opening was much smaller than, and towards one side of, the erosion.

The artery retained scarcely a drop of blood, and was of the size of a small goose's quill. There was no marked appearance of inflammation connected with the erosion, or in any other part of the stomach. There remained but little blood in the vasa brevia; yet they appeared to have been somewhat enlarged.

The small intestines were lined in many places with a greenish black matter of peculiar fœtor, in appearance resembling molasses, and adhering with great tenacity to the villous coat. A small quantity of fæces was in the colon.

The pancreas, liver, and gall-bladder were of healthy structure; in the latter was about an ounce of deep yellow bile.

The kidnies and urinary bladder seemed free of disease; the latter more than half full of urine.

The lungs adhered extensively to the pleura costalis, the effect effect of former disease; they were not tuberculous, but on being cut, gave out a brownish mucous fluid; within the cavity of the chest we found about six ounces of watery fluid.

The heart was sound, but pale, and exanguinious, a very small quantity of blood remaining in the right auricle only. About an ounce of watery fluid was within the pericardium. On cutting into the several viscera, it appeared as if little or no blood had been left in them; the vessels being almost every where nearly empty.

There was no appearance by the dissection, of any internal injury having been occasioned by the fall. And the erosion, which had so principal a share in the mischief, remains yet unaccounted for: it was said, indeed, that the subject of the case had been addicted to the use of fermented liquors; but it does not appear that he had complained of greater derangement of stomach previous to this vomiting of blood, than asthmatic persons are frequently subject to.

The hæmorrhagy itself will appear obviously to have been the consequence of the erosion. The portion of the stomach with the morbid part, which is now before us, shews some evident loss of substance of the arterial coats. It may also be remarked, that as a difficulty, more or less, of pulmonary circulation *did* exist, such an unequal distribution of the blood would the more readily take place, during a fit of coughing, as to affect for the time amongst other vessels, the cœliac artery, and its gastric branches; so that with the concurrence of this exciting cause, a still more effectual opening of the injured artery was likely to happen.

The *vomiting* of blood, it *has* been seen, ceased early in the disease, most probably because the sanguiferous system being then greatly reduced by such depletions, the supply from the bleeding artery became so much diminished, as no longer to excite the action of vomiting; while the blood, now more slowly effused, appears to have been carried *downwards*. The irreparable waste thus effected could not fail, by interrupting the functions necessary to the circulation of the blood, to be fatal to the vital principle. In the cases given by Hoffman, he explains the great prostration of strength, and fatal tendency, in such complaints, as arising rather from the putrefaction of the effused blood within the body affecting the brain, than from the *loss* of this vital fluid. (Medic. Rat. tom. 4. p. 2 s. 1. cap. 3.) This explanation, perhaps, frequently applica-

 ble, ble, appears to be more especially so in cases where the hæmorrhagy shall not have been of itself sufficiently great to account for the unfavourable issue. But, from the nature of the hæmorrhagy in the *present* case, it will appear unnecessary to seek for any *other* cause of the fatal event. At the same time, as the presence of the putrefaction alluded to, could not be doubted, it may be supposed that this did concur with the loss of blood, in occasioning the morbid symptoms.

The black coloured matter evacuated in melæna either by stool or vomiting, and sometimes both ways, is now generally supposed to consist chiefly of blood effused into the alimentary canal, and by stagnating there for a time, to be so changed, as to put on such an appearance. The presence of the atra bilis of the ancients is very rarely, if at all, any longer admitted; and Boerhaave's theory seems to be equally exploded. Yet the celebrated Morgagni, apparently in doubt upon the subject, after giving a case with the appearances on dissection, writes thus: Sed undenam tanta ista nigredo? an ab effusa in illud intestinum bile, quæ per se esset nigerrima? Videre enim potes in Cl. virorum Budæi et Schoberi observationibus fellis vesiculam magnam eadem illa materia nigricante turgidam quam ægri vomitu ejiciebant. An bilis cum antea esset subnigra, ab admistis in eo intestino certis quibusdam aliis humoribus, tanto nigrior facta est? An atri aliquid etiam a sanguine accessit, ex erosis in gravissimo illo dolore vasculis effluente? Cave enim credas eum omnem humorem sanguinem fuisse. (De sed. et caus. Morb. Ep. 30, art. 17.)

As to our particular case, a few suggestions only remain to be offered. The dissection shewed the bile of a natural colour, oozing through the common duct, towards the duodenum. But after passing, with a free admission, into the intestines, and its natural appearance lost, it is probable to suppose that it suffered some morbid change there, contaminated by the corrupted state of the effused blood; and thus possibly the bile itself promoting the general evil. Dr. Saunders, in his excellent work on the Liver, (p. 136) shews by an experiment, that the bile does not putrefy so soon as blood, each being separately exposed, in equal quantity, to the same degree of heat; but what the *reciprocal* action of these important fluids would be, from their *union* under applicable circumstances, appears to be little known.

The matter found in the small intestines, as has been seen, had not acquired so deep a dye as that which (of the the same sort) after a longer circuit through the body, came away by stool; the former had in its blackness, something of a greenish or olive hue, and was exceedingly viscid. Now, was the olive cast of this matter, as also some share of its viscid quality, owing to the admixture of bile? or, was *all* this tenacity imputable to the gluten of the effused blood? However this may be, the proportion of bile incorporated with this matter was likely to contribute to its characters in *some* way, *supposing* even the quantity of bile usually poured into the *duodenum* in a given time, according to the estimate of Valcarenghi and Haller to be much over-rated.

*Conduit Street, July* 15, 1809.

